# COVID-19 Information Dissemination Using the WeChat Communication Index: Retrospective Analysis Study

**DOI:** 10.2196/28563

**Published:** 2021-07-16

**Authors:** Zina Fan, Wenqiang Yin, Han Zhang, Dandan Wang, Chengxin Fan, Zhongming Chen, Jinwei Hu, Dongping Ma, Hongwei Guo

**Affiliations:** 1 School of Management Weifang Medical University Weifang, Shandong China; 2 "Health Shandong" Collaborative Innovation Center for Severe Social Risk Prediction and Governance Weifang, Shandong China; 3 Collaborative Innovation Center of Social Risks Governance in Health Shanghai China

**Keywords:** COVID-19, information dissemination, *People’s Daily*, Chinese news, public health and communication, media salience, WeChat

## Abstract

**Background:**

The COVID-19 outbreak has tremendously impacted the world. The number of confirmed cases has continued to increase, causing damage to society and the economy worldwide. The public pays close attention to information on the pandemic and learns about the disease through various media outlets. The dissemination of comprehensive and accurate COVID-19 information that the public needs helps to educate people so they can take preventive measures.

**Objective:**

This study aimed to examine the dissemination of COVID-19 information by analyzing the information released by the official WeChat account of the *People’s Daily* during the pandemic. The most-read COVID-19 information in China was summarized, and the factors that influence information dissemination were studied to understand the characteristics that affect its dissemination. Moreover, this was conducted in order to identify how to effectively disseminate COVID-19 information and to provide suggestions on how to manage public opinion and information governance during a pandemic.

**Methods:**

This was a retrospective study based on a WeChat official account. We collected all COVID-19–related information, starting with the first report about COVID-19 from the *People’s Daily* and ending with the last piece of information about lifting the first-level emergency response in 34 Chinese provinces. A descriptive analysis was then conducted on this information, as well as on Qingbo Big Data’s dissemination index. Multiple linear regression was utilized to study the factors that affected information dissemination based on various characteristics and the dissemination index.

**Results:**

From January 19 to May 2, 2020, the *People’s Daily* released 1984 pieces of information; 1621 were related to COVID-19, which mainly included headline news items, items with emotional content, and issues related to the pandemic’s development. By analyzing the dissemination index, seven information dissemination peaks were discerned. Among the three dimensions of COVID-19 information—media salience, content, and format—eight factors affected the spread of COVID-19 information.

**Conclusions:**

Different types of pandemic-related information have varying dissemination power. To effectively disseminate information and prevent the spread of COVID-19, we should identify the factors that affect this dissemination. We should then disseminate the types of information the public is most concerned about, use information to educate people to improve their health literacy, and improve public opinion and information governance.

## Introduction

The COVID-19 outbreak has impacted the world tremendously, and the number of confirmed cases has only continued to increase, causing damage to society and the economy worldwide [1,2]. The public pays close attention to information about the epidemic and learns about the disease through various media outlets [3]. However, as the internet develops, the spread of pandemic-related information continues to become faster and more complex [4-6]. On the one hand, the dissemination of comprehensive and accurate COVID-19 information that the public needs helps to educate people so they can take preventive measures [7]. The Singapore government, for example, immediately established a national WhatsApp account during the outbreak’s early stage to inform people living in Singapore about relevant government updates and initiatives. Over 635,000 people subscribed to the channel to receive these messages [8]. In another example, a Vietnamese dancer choreographed a dance about how to carefully wash one’s hands and even started a dance challenge on TikTok. This dance challenge video went viral, resulting in millions of people learning about essential handwashing steps. The dissemination of this information played a critical role in fighting against the spread of COVID-19 [9]. On the other hand, the spread of rumors and false information is also accelerating [10,11]. If we cannot provide timely knowledge to effectively guide and manage the spread of public opinion and rumors on the internet, the consequences will be disastrous [12]. Therefore, it is necessary to research and analyze the dissemination of COVID-19 information in order to help control the disease and disseminate pandemic-related information that the public needs, while ensuring its correct and comprehensive transmission (ie, preventing deviations, omissions, and even errors in the transmission process) [13-15]. In this era of globalization, it may be difficult to prevent the spread of COVID-19, but the most effective way to prevent panic among people is to provide reliable information that meets the public’s needs [16], information from a scientific viewpoint with a preventive attitude toward COVID-19 [17]. The first step in this effort is to study communication related to COVID-19.

WeChat is an active and important app in China [18]. WeChat official accounts disseminate information quickly and conveniently. They are, therefore, a significant means of disseminating health information [19,20]. To evaluate and measure the dissemination effect of WeChat official accounts, Qingbo Big Data Technology Co Ltd in Beijing, China, has researched and developed the WeChat Communication Index (WCI). Qingbo Big Data Technology Co Ltd is a company that provides research and development of new media big data evaluation systems, influence standards, and other big data services. It serves the Chinese government as well as China’s top news media outlets (eg, Xinhua News Agency, *People’s Daily*, and China National Radio), including internet companies [21]. This company has a respectable reputation, and the WCI that it developed has become one of the most rigorous standards for measuring the disseminating power of WeChat [22,23]. The WCI contains four main indicators concerning spread rates (ie, the spread rate of the whole article, the average spread rate of each article, the title spread rate, and the peak spread rate), eight secondary indicators, and a set of calculation formulas for the standardized scores. Specifically, the higher the WCI value, the better the dissemination effect. During the COVID-19 outbreak in China, the WCI of the *People’s Daily* WeChat official account was ranked the highest among all the WeChat official accounts. This is also China’s most representative official media [24]. Thus, it is an important channel for the Chinese people to obtain official COVID-19 information [25]. Qingbo Big Data not only recorded all of this information but also used the WCI formula to calculate and publish the dissemination index for each piece of information. By analyzing these pieces of information and their dissemination indices, we can determine significant details concerning the spread of COVID-19 information, especially the trend and influencing factors of COVID-19 information dissemination, which has important value for public health. This study contributes to the dissemination of COVID-19 information in a scientifically accurate manner and to helping people receive public health information more effectively, so they may be able to take preventive measures and control the spread of COVID-19. Moreover, in the era of globalization with the rapid development of the internet, this research can also provide a reference for relevant research in other countries using data from social media platforms, such as Twitter and Facebook. Additionally, this study can provide a reference for other countries to understand the trend of human behavior when consuming COVID-19 information, which is conducive to understanding public opinion during a pandemic, enhancing the ability of information governance during public health emergencies.

The aim of this study was to examine the dissemination of COVID-19 information. This included analyzing the status of the information released by the *People’s Daily* official WeChat account. This took place during the pandemic, while researching the dissemination trend of China’s COVID-19 information. Additionally, it involved analyzing the influencing factors and the key elements of COVID-19 information dissemination, while discussing information governance during a pandemic.

## Methods

### Data Collection

This study’s data were derived from Qingbo Big Data’s platform, which includes all COVID-19 information released by the *People’s Daily* official WeChat account and the dissemination index of each piece of information. The research cycle covered a total of 105 days, exporting all COVID-19 information from January 19, 2020, which is when the *People’s Daily* first released COVID-19 information, to May 2, 2020, which is when China’s 34 provinces (ie, autonomous regions and municipalities) completely lifted the first-level response to public health emergencies [26]. This period was chosen because it covers the entire duration of the COVID-19 pandemic, from the mainstream media’s first report to the subsequent development and normalization of the COVID-19 pandemic. The public experienced *information scarcity*, caused by few media reports being released at first, which was followed by an *information explosion* due to the media’s collective focus. Finally, a public opinion vortex was caused by the surplus of COVID-19 information [27]. This period comprehensively covers the whole process of COVID-19’s information dissemination, including each stage of communication [28].

### Inclusion and Exclusion Criteria

This study included information that was related to COVID-19 and published within the study’s established period. Any information that did not meet these criteria was excluded. All the information about COVID-19 that was released by the official WeChat account of the *People’s Daily* was included in this study. Specifically, out of the 1984 pieces of information that the account released, 1621 items were related to COVID-19.

### Statistical Analysis

This study used SPSS, version 21.0 (IBM Corp), to conduct its statistical analyses. Descriptive analysis was used to describe the basic characteristics of the COVID-19 information that was released by the *People’s Daily*, and it describes the information content with a higher dissemination power, according to the dissemination trend chart in the Results section. A multiple stepwise regression analysis was used to analyze the factors that affected COVID-19 information dissemination. Dummy variables were manually established for the multi-categorical disordering of variables, where the dummy variables were grouped under the same factor in the same block, then “ENTER” was selected as part of the inclusion method to ensure that these dummy variables entered and exited at the same time. Other continuous variables and binary variables were grouped into another block, and the inclusion method was “STEPWISE.” Then, a multiple linear regression was performed after each setting. The model’s goodness of fit was checked as well as whether there was a collinearity problem.

### Ethics Approval

As per the protocol at Weifang Medical University, China, the Institutional Review Board does not review studies that do not involve human subjects. As this study did not include human subjects, ethics approval was not required.

### Data Sharing

The data sets used and analyzed during this study are available from the corresponding author on reasonable request.

## Results

### COVID-19 Information Released by the People’s Daily

From January 19 to May 2, 2020, the *People’s Daily* released 1621 pieces of information related to COVID-19 (Table 1). In terms of media salience, this included 1129 headline news items (69.65%) and 492 nonheadline items (30.35%), which refer to prominence. This can also be divided into 1023 emotional information items (63.11%) and 598 neutral information items (36.89%), which refer to valence. The number of words for each information item was primarily between zero and 500. In terms of content, the main theme concerned the development of COVID-19 (410/1621, 25.29%). Meanwhile, the sources were primarily government agencies (1478/1621, 91.18%), and most of the information was original content from the *People’s Daily*, including 923 items (56.94%). In terms of the information’s format, the release time was primarily between 6:01 AM and 6 PM, and it was mostly presented as *text + pictures* (957/1621, 59.04%). Furthermore, the number of fonts and colors ranged between 1 and 6.

**Table 1:** Characteristics of COVID-19 information released by the *People’s Daily*.

Dimensions and variables			Categories		Information items (N=1621), n (%)		WeChat Communication Index, mean (SD)
**Media salience**							
	**Prominence**						
		Headline news items		1129 (69.65)		90,457.35 (3744.00)	
		Nonheadline items		492 (30.35)		86,747.28 (1694.76)	
	**Valence**						
		Emotional information items		1023 (63.11)		89,765.96 (3898.38)	
		Neutral information items		598 (36.89)		87,534.51 (2204.58)	
	**Attention (number of words per piece of information)**						
		0-500		1005 (62.00)		89,467.22 (3700.27)	
		501-1000		344 (21.22)		88,061.71 (3231.44)	
		>1000		272 (16.78)		88,988.84 (3540.65)	
**Informational content**							
	**Theme**						
		Policy and planning		241 (14.87)		88,620.69 (3110.21)	
		Treatment and research		230 (14.19)		90,383.53 (4233.89)	
		Initiative and mobilization		148 (9.13)		90,527.20 (3977.95)	
		Stories about fighting COVID-19		294 (18.14)		90,492.20 (3624.73)	
		Current prevalence status		410 (25.29)		86,919.27 (1909.89)	
		Epidemic prevention knowledge		270 (16.66)		89,448.48 (3675.55)	
		Other themes		28 (1.73)		88,436.71 (2766.13)	
	**Source**						
		Government		1478 (91.18)		88,861.01 (3455.07)	
		Civil organization		2 (0.12)		91,606.40 (6029.22)	
		Enterprise		104 (6.42)		90,943.57 (4243.16)	
		Personal		28 (1.73)		92,992.48 (4356.11)	
		Medical institution		5 (0.31)		92,970.53 (5142.01)	
		Research organization		2 (0.12)		89,373.20 (4850.61)	
		Other sources		2 (0.12)		93,718.10 (8885.36)	
	**Originality**						
		Original		923 (56.94)		89,143.03 (3434.57)	
		Nonoriginal		698 (43.06)		89,016.80 (3854.00)	
**Format**							
	**Time of release**						
		12:01 AM-6 AM		5 (0.31)		91,673.75 (4746.00)	
		6:01 AM-12 PM		695 (42.87)		88,494.94 (3230.48)	
		12:01 PM-6 PM		521 (32.14)		89,622.55 (3909.18)	
		6:01 PM-12 AM		400 (24.68)		89,392.62 (3710.13)	
	**Vividness**						
		Text		241 (14.87)		86,929.55 (2236.30)	
		Text + pictures		957 (59.04)		88,964.66 (3614.09)	
		Text + video		164 (10.12)		90,508.84 (3821.82)	
		Text + pictures + video		259 (15.98)		90,656.73 (3444.72)	
	**Number of fonts**						
		1 or 2		689 (42.50)		89,066.59 (3456.92)	
		3 or 4		908 (56.01)		89,058.91 (3717.90)	
		>4		24 (1.48)		90,849.01 (4173.84)	
	**Number of colors**						
		1 or 2		1360 (83.90)		88,903.74 (3579.23)	
		3 or 4		252 (15.55)		90,090.93 (3721.28)	
		>4		9 (0.56)		88,972.38 (2491.80)	

### Analysis of Informational Trends in the People’s Daily

**Figure 1:**
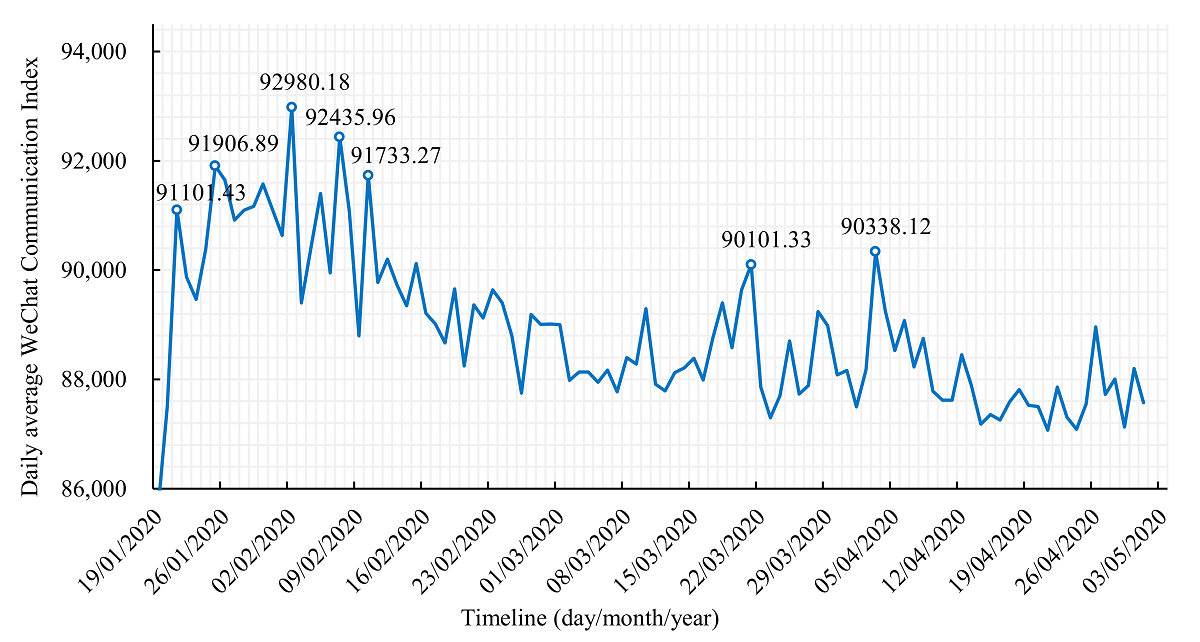
Dissemination of COVID-19 information over time.

During the COVID-19 epidemic period, the *People’s Daily* released several pieces of information related to COVID-19 every day, and the dissemination index for each piece of information represents its dissemination effect. Based on the time series analysis of the average dissemination index for all of the information released daily, we created a chart depicting the information dissemination trends. According to this chart, there were seven peaks of information dissemination from January 19, 2020, to 12 AM on May 2, 2020 (Figure 1). The analysis of these peaks and the content related to key events that occurred during these peaks revealed numerous insights.

The first peak in the dissemination of COVID-19 information occurred around January 21 (mean dissemination index of 91,101.43). The *People’s Daily* and other media began to report on COVID-19, which aroused public attention; in addition, the National Health Commission released the first announcement in 2020, which categorized COVID-19 as a Class B infectious disease, while adopting management measures as per the guidelines for more serious Class A infectious diseases. Specifically, when Zhong Nanshan confirmed that COVID-19 could be transmitted from person to person, the first peak of information dissemination was triggered. The second peak occurred around January 26, when the news reported that many new coronaviruses had been found through an epidemiological retrospective analysis [29]. The third peak occurred on February 2, when the news reported that the construction of Huoshenshan Hospital had been completed and the hospital was taken over by the Chinese People’s Liberation Army. The dissemination index on that day was 92,980.18. The fourth peak of information dissemination occurred on February 7, when Li Wenliang passed away, arousing strong public concern. The *People’s Daily* released five news items to commemorate him, and the State Supervision Commission appointed a team to investigate his death. The fifth peak happened on February 10, when the medical team’s support for Wuhan reached its peak, and nearly 6000 medical workers arrived in Wuhan, the largest number of medical staff to arrive in Wuhan since the start of the COVID-19 epidemic. The sixth peak occurred on March 21, when no new cases had been reported in either Wuhan or the Hubei Province for the first time. In fact, there were no increases in cases for the first time in 31 provinces or in Xinjiang Production and Construction Corps. The news that the epidemic situation had improved aroused public concern. The seventh peak occurred around April 3, which was a period of national mourning. The *People’s Daily* released some messages in memory of the dead, which triggered an emotional resonance among the public and led to a peak of information dissemination (mean dissemination index of 90,338.12).

Since then, the information dissemination index gradually decreased. Experts on infectious diseases and other public health emergencies found that the public have a strong curiosity and demand for information when they have a low awareness of diseases [30]. The distribution of peaks of information dissemination during this time reflects the public’s psychological needs during an epidemic, illustrating that the public will seek the truth about an event at its early stage. At the middle stage, they will question some of the events that occurred, and at the epidemic’s late stage, due to its normalization, the public’s concern will gradually decline. The whole process presents a regular development trend [31].

### Analysis of Factors Affecting the Dissemination of COVID-19 Information

The dimensions of media salience, information content, and format contained 10 factors. A multiple stepwise regression analysis was conducted with these 10 factors serving as independent variables and the dissemination indices as dependent variables. We found that eight variables entered the equation: prominence, valence, attention, theme, source, vividness, originality, and the number of fonts (Table 2). As the tolerance of the equation was greater than 0.1 and the variance inflation factor was less than 10, we could determine that no collinearity existed between independent variables (ie, the credibility of the multiple linear regression was high) [32]. As shown in Table 2, in terms of media salience, the dissemination of headline news items was better than that of nonheadline items, while the dissemination of emotional information was better than that of neutral information. Further, the fewer words that a piece had, the better its dissemination. In terms of informational content, the following variables affected the dissemination of COVID-19 information (*P*<.05): theme, source, and originality. In terms of the information’s format, its vividness affected its dissemination; specifically, this pertains to whether information was presented by text, picture, or video. The number of fonts utilized also affected dissemination, as more font variety equated to a superior dissemination effect. However, the release time and number of colors had no significant impact on the dissemination of COVID-19 information.

**Table 2:** Analysis of factors affecting the dissemination of COVID-19 information.

Dimensions and variables					Regression coefficient (SE)			Standardized regression coefficient			*t* value			*P* value
**Media salience**														
	Constant			64,378.147 (10,962.848)						5.872			<.001	
	Prominence			2150.089 (185.234)			0.273			11.607			<.001	
	Valence			2523.999 (199.787)			0.336			12.633			<.001	
	Attention			–0.151 (0.071)			–0.048			–2.122			.03	
**Informational content**														
	**Theme (other theme = 0)**													
		Policy and planning	1121.800 (593.446)			0.110			1.890			.06		
		Treatment and research	2079.100 (592.525)			0.200			3.509			<.001		
		Initiative and mobilization	1777.394 (610.512)			0.141			2.911			.004		
		Stories of fighting COVID-19	1253.960 (584.977)			0.133			2.144			.03		
		Current prevalence status	421.651 (592.767)			0.051			0.711			.48		
		Epidemic prevention knowledge	1641.571 (592.536)			0.169			2.770			.006		
	**Source (other sources = 0)**													
		Government	4943.180 (2090.008)			0.387			2.365			.02		
		Civil organization	3286.498 (2949.465)			0.032			1.114			.27		
		Enterprise	4174.864 (2104.164)			0.283			1.984			.047		
		Personal	2238.679 (2160.768)			0.081			1.036			.30		
		Medical institution	3091.545 (2467.104)			0.047			1.253			.21		
		Research organization	5957.447 (2946.397)			0.058			2.022			.04		
	Originality			481.217 (201.708)			0.066			2.386			.02	
**Format**														
	**Vividness (text = 0)**													
		Text + pictures	772.974 (265.608)			0.105			2.910			.004		
		Text + video	788.653 (366.266)			0.066			2.153			.03		
		Text + pictures + video	758.213 (332.765)			0.077			2.279			.02		
	Number of fonts			318.600 (90.585)			0.096			3.517			<.001	

## Discussion

### Principal Findings

Based on results of the WCI, this study examined the COVID-19 information that was first spread by the *People’s Daily*. This was conducted by analyzing the information’s media salience, content, and format. Thereafter, we summarized the COVID-19 information that was popular during the pandemic period. Seven information dissemination peaks were discerned. We further analyzed the influencing factors and key elements of COVID-19 information dissemination. We found that among the three dimensions of COVID-19 information—media salience, content, and format—eight factors affected the spread of COVID-19 information.

During the COVID-19 pandemic period, most of the information released by the *People’s Daily* focused on the pandemic’s development, with daily announcements about the number of confirmed cases, which kept the public abreast of related developments and helped dispel rumors. The public WeChat account of the *People’s Daily* mostly took the form of headlines upon releasing COVID-19 information, and most items had positive emotional tendencies. It seems that extensive positive reports played a role in stabilizing people’s emotions [33]. Most of the information came from the national and local health commissions, which ensured that the *People’s Daily* portrayed authority and fairness [34]. The *People’s Daily* is the most authoritative official media channel in China and, as such, the WCI of its official WeChat account ranked the highest during the epidemic. It was the responsibility of official media outlets to release relevant information about COVID-19 in a timely manner to spread relevant knowledge. This helped the public to have an awareness of the pandemic and improved the situation in China [35]. This shows the network media’s power when it comes to disseminating COVID-19 information [36].

By analyzing the trend regarding the average daily dissemination index of information, as well as content and key events, we found that there were seven peaks of information transmission during the COVID-19 epidemic period, which began on the following dates in 2020: January 21, January 26, February 2, February 7, February 10, March 21, and April 3. These peaks were primarily concentrated on the following issues: the media’s first reported coverage about COVID-19, epidemiological studies that confirmed the existence of human-to-human disease transmission, and government measures to manage the epidemic. These findings are consistent with research conducted by Liu Lanlan, who found that the dissemination peaks corresponded to January 26 and other dates similar to those found in this study, and information mainly concentrated on Zhong Nanshan’s confirmation of COVID-19’s transmission via person-to-person contact and Li Wenliang’s commemoration, among other key events [31]. The WCI’s peak value shows where the public’s attention was focused and their psychological need for epidemic information. Thus, the mainstream media should release COVID-19 information in a targeted manner and disseminate the type of information that the public values the most [37]. It should also improve the timeliness of information release, educate people on preventive measures more effectively, and improve people’s health literacy so they can better manage COVID-19 [38].

Our research shows that media salience, information content, and format affect COVID-19 information dissemination, specifically in terms of eight variables: prominence, valence, attention, theme, source, vividness, originality, and number of fonts. However, the time that the information was released and its number of colors had no effect on dissemination. These results suggest that different types of epidemic information disseminate differently; therefore, we must consider the factors affecting information dissemination and choose the best dissemination practices during the process of COVID-19 prevention and control to ensure that information is spread effectively and in a responsible manner. This would thereby prevent the spread of rumors and false information, improve the information’s scientific and authoritative reputation [39], increase the ability to manage information and public opinion during epidemics, and contribute to a comprehensive public health system [40].

### Strengths and Limitations

This study has several strengths regarding its design and analysis. First, the selected research objects were relatively representative. The WCI of the *People’s Daily* official WeChat account showed that it was top-ranked among all WeChat official accounts, and this account is an important channel for the Chinese people to obtain official COVID-19 information. Second, based on big data technology, the WCI obtains data from all WeChat users (more than one billion) [41], resulting in a large sample size and high accuracy; therefore, the research findings are representative. Finally, the findings have practical significance; for example, this study found that headline news item dissemination has more impact than nonheadline items. These findings could inform the accurate dissemination of COVID-19 information and knowledge in the future. However, several limitations should be considered when interpreting the results. Because of the large number of potential factors and limited related research, we selected the three most commonly used dimensions—constituting 10 variables—from existing research, which may limit the research results. In addition, this study mainly analyzed the most popular social media platform in China. Although this study serves as an example for other countries to study COVID-19 information dissemination, it may not be completely applicable to other countries. This limits the inferences one may make from these results. Future studies can use more comprehensive potential factors to verify or enrich the conclusions of this study.

### Conclusions

Currently, only a few indicators have been explored to quantify the effect of COVID-19 information dissemination. It is difficult to measure the spread of information when using quantitative data. It is even more complex as it occurs in conjunction with the unexpected and uncertain nature of COVID-19. This leads to only a few studies in the literature that have attempted to understand the effect and measurement of COVID-19 information dissemination. Thus, to fill this gap, we measured the dissemination of COVID-19–related information using WCI data, integrating the areas of public health and communication science (ie, media salience), to determine the factors affecting information dissemination. This study showed that the different types of pandemic-related information had varying dissemination power. It also revealed the factors that affected this dissemination: media salience, information content, and information format. These findings suggest that for effective dissemination of COVID-19 information, we should pay attention to the factors that affect this dissemination, which include the theme, source, and originality of COVID-19 information. Furthermore, we should disseminate the type of information that the public is most concerned about, choose those dissemination practices that effectively spread information in a responsible manner to curtail misinformation, regulate fear among the public, and improve information governance. Further research is needed to confirm the findings of this study and to validate the impact of these findings on the effect of COVID-19 information dissemination in other countries.

## Abbreviations

WCI: WeChat Communication Index
